# Valvular Regurgitation and Congestive Heart Failure in an Elderly Dutch Rabbit (*Oryctolagus cuniculus*)

**DOI:** 10.1155/crve/9959506

**Published:** 2025-12-28

**Authors:** Nooshin Derakhshandeh, Amirreza Hashemian, Saghar Karimi

**Affiliations:** ^1^ Department of Clinical Sciences, School of Veterinary Medicine, Shiraz University, Shiraz, Iran, shirazu.ac.ir

**Keywords:** congestive heart failure, mitral valve regurgitation, rabbit, tricuspid valve regurgitation

## Abstract

Advances in veterinary diagnostics and treatment have increased awareness of cardiovascular diseases in pet rabbits, with age‐related conditions such as valvular disorders becoming more common as these animals live longer. This report describes a seven‐year‐old male Dutch rabbit (*Oryctolagus cuniculus*) diagnosed with congestive heart failure caused by both mitral and tricuspid regurgitation. Clinical signs included coarse lung crackles, mild tachycardia, and muffled heart sounds. Blood analysis showed moderate leukocytosis with neutrophilia and monocytosis. Echocardiography revealed enlargement of the left atrium, significant valvular regurgitation, and small fluid accumulations in the pleural and pericardial cavities. Treatment with furosemide and enalapril provided brief improvement over 2 weeks before the rabbit′s death. The case highlights valvular regurgitation as a frequent cause of heart failure in aging rabbits and emphasizes the value of early detection and timely therapy in improving outcomes and extending quality of life in affected animals.

**Summary**

• Valvular heart disease is increasingly recognized in elderly companion rabbits due to advances in longevity, diagnostics, and awareness.
• Mitral and tricuspid regurgitation are important causes of congestive heart failure in this species, often presenting late due to rabbits′ ability to mask clinical signs.
• Early detection is challenging—subtle findings such as muffled heart sounds, coarse crackles, or mild tachycardia warrant thorough cardiovascular evaluation.
• Echocardiography is indispensable for noninvasive, accurate diagnosis of valvular pathology, cardiac chamber enlargement, and associated effusions in rabbits.
• Prompt medical management may yield temporary improvement, but prognosis for advanced valvular insufficiency remains guarded.
• Owner education on early clinical signs can facilitate earlier presentation and intervention, potentially improving quality of life and survival time.

## 1. Background

In this case, an elderly pet rabbit developed congestive heart failure caused by both mitral and tricuspid valve regurgitation. The problem was identified and treated successfully without the need for invasive procedures. Heart disease in rabbits, especially those living as pets, has rarely been documented, and because these animals often hide signs of illness, it is usually detected late in its course.

## 2. Case Presentation

A seven‐year‐old, intact male Dutch rabbit (*Oryctolagus cuniculus*) was presented to the clinic following a 3‐week history of abdominal bloating, poor appetite, low energy, diarrhea, and noticeable weight loss. The owner noted that the rabbit had not urinated at all in the 24 h before arrival. On examination, the animal was weak and quiet but remained alert. Listening to the chest revealed coarse crackling sounds in the lungs, a slightly rapid heartbeat at over 340 beats per minute (normal range: 198–330 bpm) [1], and unusually muffled heart sounds.

## 3. Investigations

**Figure 1:**
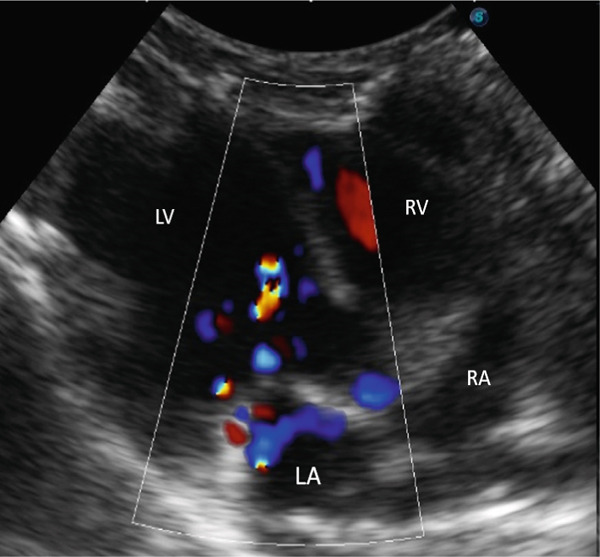
Right parasternal longitudinal 4‐chamber echocardiogram shows moderate mitral valve regurgitation (blue signals in left atrium in systole with closed valves). LA: left atrium, LV: left ventricle, RA: right atrium, and RV: right ventricle.

**Figure 2:**
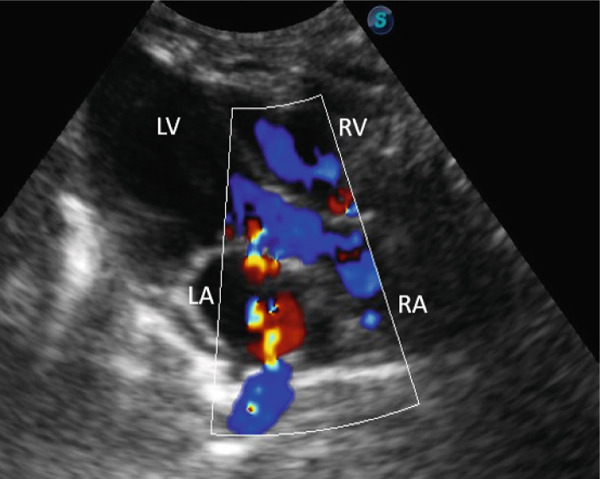
Right parasternal longitudinal 4‐chamber echocardiogram shows moderate tricuspid valve regurgitation (blue signals in right atrium in systole). LA: left atrium, LV: left ventricle, RA: right atrium, and RV: right ventricle.

**Figure 3:**
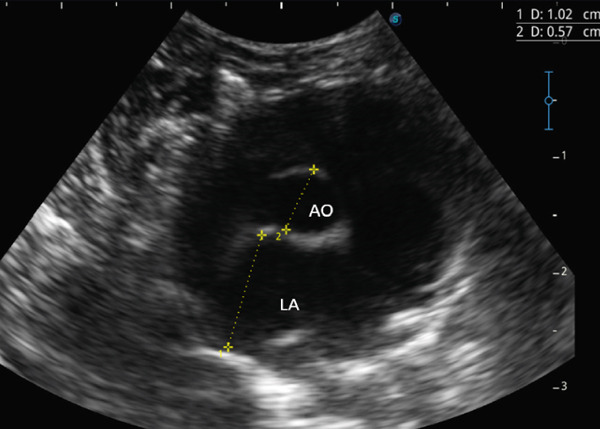
Right parasternal short‐axis view of the aorta and left atrium. Number 1 shows the left atrium diameter (1.02 cm) and Number 2 shows the aortic diameter (0.57 cm). AO: aorta and LA: left atrium.

Additional findings included poor body condition, pale mucous membranes, and a prolonged capillary refill time [2]. Laboratory analysis demonstrated mild leukocytosis (11.1 × 10^3^/*μ*L; reference range: 6.30–10.06 × 10^3^/*μ*L), neutrophilia (6.21 × 10^3^/*μ*L; reference range: 1.49–3.21 × 10^3^/*μ*L), and moderate monocytosis (1.11 × 10^3^/*μ*L; reference range: 0.05–0.45 × 10^3^/*μ*L) [2]. Thoracic and abdominal radiographs confirmed pleural effusion and a bronchoalveolar pattern in the caudodorsal lung fields, consistent with pulmonary edema, whereas abdominal structures appeared normal apart from the presence of bladder sludge. Echocardiography revealed moderate regurgitation of both the mitral and tricuspid valves, enlargement of the left atrium, increased thickness of the left ventricular free wall during diastole, and mild pleural effusion (Figures 1, 2, and 3; Table 1). Thoracocentesis yielded serosanguineous fluid which, upon analysis, was classified as a modified transudate (protein concentration: 22.2 g/dL; reference range: 25–30 g/dL; nucleated cell count: 2000/*μ*L; reference range: 1000–7000/*μ*L) [2].

**Table 1:** This table shows echocardiographic values from the case and reference range values where they have been established from 100 New Zealand white rabbits [3].

**Echocardiographic parameters**	**Case**	**Reference range**
Aorta (mm)	5	6.39–9.41
Left atrial dimension diastole (mm)	10.2	6.62–10.62
Left atrium/aorta	2	0.89–1.29
Thickness of the interventricular septum in diastole (mm)	1.6	1.74–3.74
Thickness of the interventricular septum in systole (mm)	2	2.64–5.38
Left ventricular internal diameter in diastole (mm)	10.3	9.54–17.02
Left ventricular internal diameter in systole (mm)	8.3	5.44–11.20
Thickness of the left ventricular free wall in diastole (mm)	5.1	1.72–3.84
Thickness of the left ventricular free wall in systole (mm)	5.2	2.54–4.58
Fractional shortening (%)	23	27.39–46.95
Ejection fraction (%)	52	58.73–83.51

## 4. Differential Diagnosis

### 4.1. Treatment

Treatment with furosemide and enalapril led to transient improvement over 2 weeks before the patient′s death.

### 4.2. Outcome and Follow‐Up

The patient died 2 weeks later.

## 5. Discussion

In recent years, cardiovascular disease in pet rabbits has been identified with increasing frequency, reflecting advances in both diagnostic and therapeutic capabilities [4]. Despite this trend, spontaneous heart disease remains uncommon, with relatively few cases described in the literature [5–7]. Similar to other species, cardiac disorders in rabbits can arise from diverse causes, including infectious agents, exposure to toxic substances, and dietary imbalances [8]. As improvements in veterinary care have extended the lifespan of rabbits, age‐related cardiac conditions—particularly valvular disease—are being encountered more often. Cardiovascular abnormalities in this species are generally classified as valvular heart disease, cardiomyopathy, or arrhythmias [8].In the present case, the exact etiology of the cardiac pathology could not be determined. Echocardiographic examination indicated that insufficiency of both the mitral and tricuspid valves likely contributed to the heart failure and left atrial enlargement observed. These valvular defects, relatively common in domestic rabbits, may result from primary valve degeneration, cardiomyopathy, or infectious processes. Progressive deterioration of such lesions can lead to volume overload and eventual congestive heart failure [4]. The most consistent clinical sign of mitral and tricuspid regurgitation is a characteristic localized murmur, whereas overt congestive heart failure is more typically associated with pulmonary edema, pleural effusion, or hepatomegaly resulting from anatomical or functional cardiac compromise [7].Rabbits′ capacity to adjust their activity levels often conceals early disease manifestations, with diagnosis frequently occurring at advanced stages [4]. In this case, although dyspnea was absent, coarse crackles on auscultation, mild tachycardia, and muffled heart sounds were present, illustrating the subtlety with which cardiac disease can present in this species. Mitral valve regurgitation, in particular, is increasingly recognized as a prevalent condition among older rabbits. Echocardiography has become indispensable for assessing cardiac structure and function, and in this case it was pivotal in detecting mitral and tricuspid regurgitation along with left atrial enlargement and effusion. Although echocardiography enables precise, noninvasive evaluation, necropsy remains a highly specific and sensitive means of identifying the underlying cause of cardiac lesions [3, 9]. A decision was made not to perform an autopsy due to the patient′s favorable clinical response to treatment and the improvement in its condition. A key limitation of this report is the absence of histological confirmation. As the owner declined euthanasia, necropsy could not be performed. When feasible, postmortem examination incorporating both desmin immunostaining and Masson′s trichrome staining is recommended, as these techniques are considered reliable for detecting early myocardial infarction or ischemic changes. Future similar cases would benefit from obtaining tissue samples with these methods to enhance diagnostic accuracy.

## Ethics Statement

All procedures used in this study were approved by the Shiraz University, School of Veterinary Medicine ethical committee and were compatible with Directive 2010/63/EU on the protection of animals used for scientific purposes.

## Conflicts of Interest

The authors declare no conflicts of interest.

## Author Contributions

N.D.: managing and treatment of the case, data curation, review and editing, and visualization. A.H.: writing the original article and following the case. S.K.: performing the echocardiography, data curation, and review and editing.

## Funding

This study was supported by the School of Veterinary Science, Shiraz University (10.13039/100009587; 0GRC1M369708).

## Data Availability

The data that support the findings of this study are available from the corresponding author upon reasonable request.
